# Supplementary material for: Alternative splicing at neuroligin site A regulates glycan interaction and synaptogenic activity
Source: eLife. 2020 Sep 11;9:e58668. doi: 10.7554/eLife.58668 (PMC7486126; doi:10.7554/eLife.58668)
Supplement: Supplementary file 1. — Accession numbers refer to the Gene Expression Omnibus or Sequence Read Archive. Sample description ages indicate embryonic days (E) or postnatal days (P) or weeks (W). Genotypes refer to single (KO), double (DKO) and triple (TKO) knockout ESC-derived neurons or mice. Library type indicates paired end (PE) or single end (SE). [file elife-58668-supp1.docx]

**Supplementary Table 1. Summary of RNA-seq datasets analyzed in this study**

| Study | Figure | Accession number |  | Tissue | Sample description | Library type | Total read  number |
| --- | --- | --- | --- | --- | --- | --- | --- |
| Tasic et al., 2018 | 3A | GSE115746 |  | Visual cortex | 8,414 single cells (core cells) | PE 51nt | 22,790,704,728 |
| Tasic et al., 2018 | 3B | GSE115746 |  | Anterior Lateral motor cortex | 12,740 single cells (core cells) | PE 51nt | 31,047,590,020 |
| Furlanis et al., 2019 | 3C | GSE133291 |  | Cortex and hippocampus | 8 neuronal cell types, each with 4 biological replicates | PE 101nt | 8,621,488,551 |
| Yan et al., 2015 | 4A | SRP055008 |  | Cortex | 9 samples at E14, E16, P0, P7, P15, P30, P110, and 21 months, each with 2 biological replicates | PE 101nt | 3,456,180,370 |
| Lister et al., 2013 | 4B | GSE47966 |  | Frontal cortex | 7 samples at fetal stage, 1W, 2W, 4W, 6W, 10W and 22 months, each with various number of biological or technical replicates | SE 101nt | 1,882,083,523 |
| Jacko et al., 2018 | 4C | SRP128054 |  | ESC-derived motor neuron | ESC-derived motor neurons at day 5 or day 10 with *Rbfox1/2/3* TKO or not, each with 2 or 3 biological replicates | PE 101nt | 392,425,505 |
| Charizanis et al., 2012 | 4D | GSE38497 |  | Brain | WT or Mbnl2 KO brain tissues, each with 3 biological replicates | PE 40nt | 140,103,131 |
| Weyn-Vanhentenryck et al., 2018 | 4D | SRP142522 |  | Frontal cortex | WT or *Mbnl1/2* DKO frontal cortex tissues, each with 3 biological replicates | PE 101nt | 364,615,403 |
| Li et al., 2014 | 4E | GSE51733 |  | Brain and cortex | WT or *Ptbp2* KO brain or cortex tissues | PE 100nt | 657,339,603 |
